# Assembly of Nsp1 Nucleoporins Provides Insight into Nuclear Pore Complex Gating

**DOI:** 10.1371/journal.pcbi.1003488

**Published:** 2014-03-13

**Authors:** Ramya Gamini, Wei Han, John E. Stone, Klaus Schulten

**Affiliations:** 1 Beckman Institute, University of Illinois at Urbana-Champaign, Champaign, Illinois, United States of America; 2 Center for Biophysics and Computational Biology, University of Illinois at Urbana-Champaign, Champaign, Illinois, United States of America; 3 Department of Physics, University of Illinois at Urbana-Champaign, Champaign, Illinois, United States of America; Max Planck Institute for Biophysical Chemistry, Germany

## Abstract

Nuclear pore complexes (NPCs) form gateways for material transfer across the nuclear envelope of eukaryotic cells. Disordered proteins, rich in phenylalanine-glycine repeat motifs (FG-nups), form the central transport channel. Understanding how nups are arranged in the interior of the NPC may explain how NPC functions as a selectivity filter for transport of large molecules and a sieve-like filter for diffusion of small molecules (<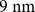 or 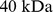). We employed molecular dynamics to model the structures formed by various assemblies of one kind of nup, namely the 609-aa-long FG domain of Nsp1 (Nsp1-FG). The simulations started from different initial conformations and geometrical arrangements of Nsp1-FGs. In all cases Nsp1-FGs collectively formed brush-like structures with bristles made of bundles of 2–27 nups, however, the bundles being cross-linked through single nups leaving one bundle and joining a nearby one. The degree of cross-linking varies with different initial nup conformations and arrangements. Structural analysis reveals that FG-repeats of the nups not only involve formation of bundle structures, but are abundantly present in cross-linking regions where the epitopes of FG-repeats are highly accessible. Large molecules that are assisted by transport factors (TFs) are selectively transported through NPC apparently by binding to FG-nups through populated FG-binding pockets on the TF surface. Therefore, our finding suggests that TFs bind concertedly to multiple FGs in cross-linking regions and break-up the bundles to create wide pores for themselves and their cargoes to pass. In addition, the cross-linking between Nsp1-FG bundles, arising from simulations, is found to set a molecular size limit of <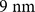 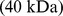 for passive diffusion of molecules. Our simulations suggest that the NPC central channel, near the periphery where tethering of nups is dominant, features brush-like moderately cross-linked bundles, but in the central region, where tethering loses its effect, features a sieve-like structure of bundles and frequent cross-links.

## Author Summary

Cells of higher life forms separate their genomes from the rest of the cell in a nucleus that surrounds the genome by a nuclear envelope. Hundreds of pores, each a complex made of many proteins, assure traffic into and out of the nucleus through highly selective transport: small biomolecules can pass unhindered, whereas large biomolecules need to associate with proteins called transport factors, to pass. Little is known about how the nuclear pore complexes function, a key impediment to observation being their huge size and the disordered nature of the pore interior. We investigated computationally what kind of structure the nuclear pore proteins (nups) form. In the computation we place many nups, each a 600 amino acid-long protein, into arrangements considered representative for the nuclear pore, and simulate the subsequent molecular behavior. We find that the nups form bundles of 2–27 proteins, the bundles being cross-linked when a single nup leaves a bundle and joins an adjacent one. The finding suggests an adaptive molecular mesh arrangement of nups in the nuclear pore and explains how selective transport is accomplished, namely that passage of sufficiently small molecules is unhindered by the cross-linking, but that large molecules need the assistance of transport factors to melt the cross-linking.

## Introduction

Nuclear pore complexes (NPCs) are large protein assemblies embedded in the nuclear membrane that provide the only conduit for exchange of molecules between cytoplasm and nucleoplasm, a process defined as nucleocytoplasmic transport (NCT). While the membrane bound nucleus of a eukaryotic cell protectively envelopes the genetic material, separating it from the cytoplasm, processes such as DNA transcription require access to the genetic material in the interior of the nucleus by a myriad of molecules, including large molecules such as proteins. Two modes exist for the NCT: passive diffusion of molecules, smaller than 40 kDa or 9–10 nm in diameter [1], and selective transport into and out of the nucleus for larger molecules such as proteins, RNA, ribosomal subunits, the larger molecules being recognized by transport factors (TFs), carrier proteins, which shuttle back and forth ferrying their cargo through the NPC.

NPCs are giant complexes having molecular masses ranging from ∼65 MDa (in yeast) to ∼125 MDa (in higher eukaryotes) that are made of copies of 30 different proteins termed nucleoporins (nups) [2]–[5]. Recently, a detailed 3D model for the position and abundance of each nup in the *Saccharomyces (S.) cerevisiae* NPC structure was proposed based on experimental data obtained from molecular, biochemical and structural studies of the NPCs and their components [6], [7]. In the modeled structure, the scaffold of the NPC is formed by two protein subcomplexes that, through linker proteins, anchor a set of FG-containing nups [6]. The complex has 8-fold symmetry about its central axis and 2-fold symmetry about the equatorial plane such that each nup is repeated 8-, 16-, 32-, or 48-fold. Altogether, the NPC forms a multi-protein complex of nearly ∼450 proteins [4]–[7].

About one-third of all pore proteins constitute the barrier proteins also known as FG-nups (F and G represent amino acids phenylalanine and glycine, respectively). FG-nups are intrinsically disordered, rich in FG-repeat motifs [4], [5], [8]–[10] and form the central transport channel of the NPC extending, however, also into the cytoplasmic and nucleoplasmic space. While the FG-nups extending toward cytoplasm and nucleoplasm sides are asymmetric in distribution, the FG-nups (eg., Nsp1) of the central region have copies distributed symmetrically around the scaffold of the NPC and are critical for bidirectional NCT. The FG-nups are present in different lengths (a few to several hundred amino acids long) and exhibit different amino acid composition and repeat motifs, typically FG, GLFG, or FxFG motifs (x being any amino acid, largely serine) separated by linker regions of 10–20 hydrophilic amino acids [4], [9], [10]; their different spatial localizations render the central channel heterogeneous. These nups play a central role in selective transport of the NPC and this selectivity is attained by bearing specific binding sites (the FG motifs) for the TFs that undergo multiple, low-affinity interactions with nups as they permeate their way through the channel [11]–[17].

Given that the central transport channel is filled with heterogeneous and natively unstructured FG-nups that are not susceptible to crystallization for structure determination, the important question how the unstructured FG nups are assembled in the interior to promote efficient and selective gating could not be answered unequivocally.

Several models have been proposed so far to explain the structure of the interior of the NPC. According to the virtual gate model [4], [18], the tethered, unstructured FG-repeat proteins form an entropic barrier that repels non-specific cargo, preventing it from reaching the interior of the channel, whereas TFs that interact with the FG-repeats of nups in the NPC interior channel have higher probability of entering and permeating the channel.

An alternative model, namely the polymer brush model [19], [20], is based on the above model suggesting that the FG-nups form extended brush-like polymers with inter-linked bristles that reversibly collapse upon binding of TFs. TFs pass the channel by repetitive binding and unbinding to the nups' FG repeats until they reach the exit of the pore [21].

In contrast, the selective phase/hydrogel model [22] proposes that FG nups in the central channel achieve a sieve-like meshwork through weak inter-repeat FG-FG hydrophobic interactions to form a hydrogel within the central channel [23]. TFs bind to the FG-repeats via low-affinity interactions which transiently melt these FG-FG cross-links of the meshwork, allowing thus only TFs to permeate the channel. Furthermore, non-specific cargo that cannot bind is filtered out [23], [24].

Another model, the reduction of dimensionality (ROD) model, proposes that the TFs act as ferries, carrying cargo and sliding on the surface of phenylalanine-glycine (FG) motifs by interacting with the FGs according to a 2D random walk rather than a 3D diffusion [25].

The ‘forest’ model, later modified to the tube gate model [26], [27], proposes the interior of the NPC to be a hydrogel and the periphery to be brush-like, featuring two separate zones of traffic with distinct physiochemical properties. In their study, based on the hydrodynamic radius of individual nups localized in different regions of the NPC, the respective authors classify nups as (a) ‘shrubs’ that form cohesive domains with collapsed-coil and low charge content; (b) ‘extended coils’ that form high-charge content, non-cohesive domains; (c) ‘trees’ having features of both (a) and (b). Thus, a ‘forest-like’ structure of FG-nups is formed in the NPC. In this model, the collapsed-coil domains are hypothesized to form a transport zone 1 in the central pore and the extended-coil domains form a peripheral zone 2. The forest model is based on the observations of individual nup conformations, not considering interactions between nups.

In a recent study on the role of nups in NPC gating, Tagliazucchi et al [28] deduced a potential of mean force of TF transport from the amino acid (positive, negative, hydrophobic) distribution and claimed that this potential can explain the use of TFs for traffic across the nuclear envelope. The claim is based, however, on the assumption that nups are individually random and do not form any structures in the channel interior as suggested by the earlier models of the NPC interior discussed above. The authors also do not attribute an explicit role to TFs interacting with nups through FG motifs.

Most models propose an FG network as the main mechanism for pore selectivity and address to a much smaller degree a role for the actual molecular structures formed by the disordered nups of the NPC. Likely, the qualitative differences of the models can be reconciled with the heterogeneity found along entire transport pathways through the NPC that furnishes different environments on the cytoplasmic side, in the interior and on the nucleoplasm side of the NPC.

To identify computationally the structure formed by the nups in the interior of the NPC pore, Miao and Schulten [20] carried out molecular dynamics (MD) simulations of nup polymers, using coarse-grained MD. Unlike the other approaches arriving at models for NPC gating, their approach included details of individual amino acids and, more importantly, included the interactions between nup polymer chains. In the simulations these chains were end-grafted to a flat surface in a 5×5 array. The MD simulations suggest that the nups have a strong tendency to form bundles of typically 2–6 proteins and that the bundles are inter-linked in a mesh-like manner as single nups cross frequently from one bundle to another one. From this behavior results a brush structure that is made of bundles of interlinked bristles. This structure can explain to some degree the gating of NPC pores: the structure can be passed by small biomolecules readily while larger biomolecules need to alter the structure to pass through. This role, namely of cutting through the mesh-like structure, could be played by transport factors.

While the structure model of Miao and Schulten is highly suggestive, the study had obvious shortcomings imposed at the time by limitations in available computer power. One shortcoming is the short length (100-aa) of nups employed that is in contrast to the actual, much longer (about 600-aa) lengths of many nups. A second shortcoming is that only a single type of nup tethering was investigated, namely tethering to a flat surface. A third shortcoming is that the simulated ensemble started with all nups initially in a highly ordered, namely fully-extended, straight chainconformation.

Enjoying today in the form of petascale computers tremendously more computer power than was available to Miao and Schulten we have performed for the present study microsecond-long simulations on various conformational ensembles of the 609-aa-long FG domain of Nsp1 (hence termed Nsp1-FG), a key nup in the NPC central channel. Three different ensembles were investigated, namely ensembles of untethered Nsp1-FGs, of Nsp1-FGs tethered to a flat surface as in the Miao and Schulten study, and of Nsp1-FGs tethered to a ring-like surface that is qualitatively similar to the geometry of the NPC. Most essential, however, is that the present study explores Nsp1-FG ensembles in different initial protein conformations, namely fully-extended conformations as assumed by Miao and Schulten as well as random polymer-like conformations. Nevertheless, the new MD simulations support the structural model as seen in the simulations of Miao and Schulten, in which 100-aa-long fragments of Nsp1-FG form bundles linked through single proteins crossing between bundles, but reveals more physical characteristics and a high degree of heterogeneity of the model. For example, the new and more extensive simulations demonstrate that the actual cross-linked bundle system depends to a significant degree on the actual tethering of nups' ends as well as on the initial nups conformation. A further key finding is that the FG motifs distributed in the cross-linking regions have highly accessible side groups that furnish potential binding epitopes for TFs to assist in cargo transport.

## Results

To elucidate how the NPC poses a barrier for transport of molecules into and out of the nucleus, we thought to characterize through simulations the general structural features in the NPC central channel, arising from the assembly of disordered proteins. We investigated the assembly using only one type of FG-nups, namely Nsp1. Nsp1 is an FxFG-rich nup present in the central channel and has been investigated as a model protein in previous studies [20], [24], [26]. The nup is present in 32 copies in both the upper and lower half of the NPC equatorial plane; its location is ∼30 nm from the center of the NPC [29]. The large number of copies and its spatial location inside the central channel renders Nsp1 critical for bi-directional nucleo-cytoplasmic transport (NCT). The 609-aa-long FG-domain of this protein, namely Nsp1-FG, was described through coarse-grained simulations that investigated the structures formed by different ensembles of Nsp1-FGs.

In two first simulations, the proteins were tethered to a gold ring surface (simulations wild-type_ring and mutant_ring). For the initial protein conformations we assumed fully-extended Nsp1-FGs as also adopted in the earlier study by Miao et al [20]. However, rather than a 100-aa-long fragment of FG-domain simulated in their study, we used in the present study the full-length (609-aa-long) FG-domain. The two simulations (wild-type_ring and mutant_ring) differ in that the first employs wild-type Nsp1-FG, whereas the second employs a mutant Nsp1-FG, with all its phenylalanines and glycines replaced by alanines. In a third simulation, Nsp1-FGs were tethered to a flat gold surface (simulation random_array). In this case the proteins were assumed initially in a random polymer conformation. In a fourth simulation the effect of end-tethering was investigated by simulating Nsp1-FGs untethered and initially homogeneously distributed in bulk solvent in random polymer conformations (simulation random_bath). All ensembles contain the protein at a concentration that is characteristic for the interior of the NPC channel. The simulations carried out are listed in Table 1.

**Table 1:** Systems simulated.

Name	Simulated system	Time (ns)	Simulated particles	System size (Å^3^)
wild-type_ring	120 fully extended, wild-type Nsp1-FGs grafted on a gold nano-pore	1000	15,453,214 CG-beads	1244×1244×1627
mutant_ring	120 fully extended, mutant (FG→AA) Nsp1-FGs grafted on a gold nano-pore	1000	16,019,434 CG-beads	1244×1244×1627
random_array	5×5 array of worm-like chain Nsp1-FGs grafted on a gold substrate	1000	1,097,433 CG-beads	290×290×1075
random_bath	120 worm-like chain Nsp1-FGs, freely floating in a water bath	1000	3,091,910 CG-beads	755×755×755
fragment_AA	11 fragments of Nsp1-FGs from the resulting final structure of simulation wild-type_ring, freely floating in a water bath	100	967,595 atoms	121×414×755

In the left column, conventional names of the simulations, as used throughout the paper, are defined. In column “Simulated particles (CG-beads or atoms)”, the total number of coarse-grained beads or atoms simulated is given, adding up particles describing protein, gold substrate and solvent (water and ions).

**Figure 1:**
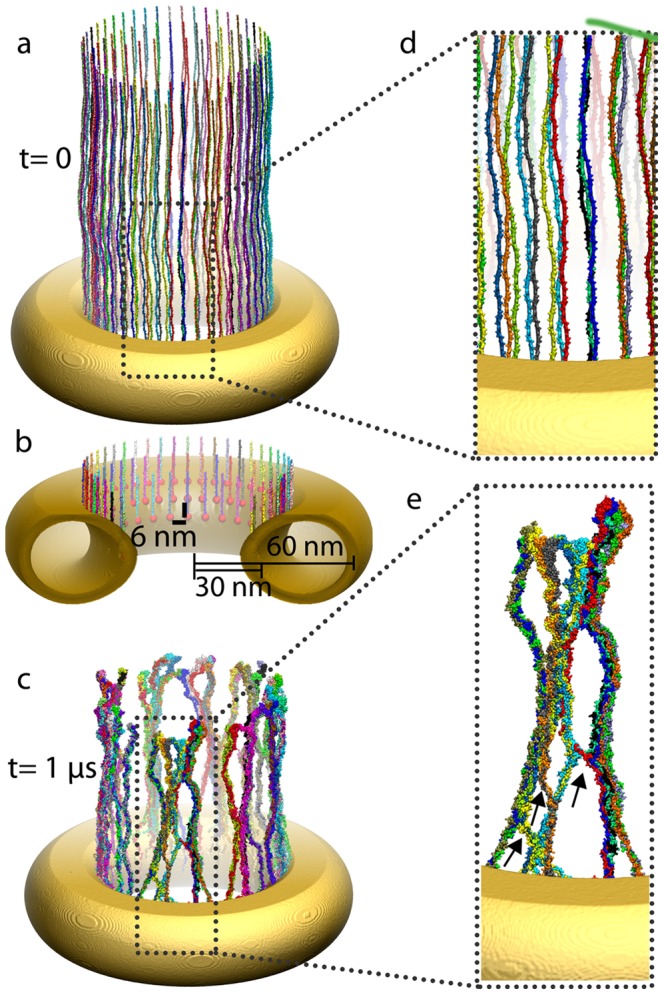
Initial and final configuration of simulated wild-type Nsp1-FGs grafted to a gold ring (simulation wild-type_ring). *(a)* Initial configuration. Shown are fully-extended, wild-type Nsp1-FGs grafted on the ring, the geometry of which matches that of an experimentally constructed nanodevice mimicking an NPC as reported in [21], [29]. Colors distinguish 120 wild-type Nsp1-FGs grafted on the ring in three concentric rows. *(b)* Close-up view of grafted ends of the Nsp1-FG chains. The gold nano-ring is cut open to expose the C-termini, shown as red spheres, fixed to the gold ring, as well as the terminal parts of the Nsp1-FG chains. *(c)* Snapshot of the (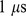) end of simulation wild-type_ring. One can recognize that the Nsp1-FG chains, shown in surface representation, have formed brush-like bundles. *(d)* Close-up view of the structure in *(a)*. Shown is a region as marked. The close-up view reveals the initially straight conformation of the Nsp1-FG chains; bumps in the surface of the individual chains correspond to amino acid side groups. *(e)* Close-up view of a segment of *(c)*. The view reveals the brush-like bundles formed by the Nsp1-FG chains. Arrows point to cross-links between bundles formed when single Nsp1-FG chains cross from one bundle to another bundle. As a result of such cross-links the bundles form a mesh of thick (bundles made of several Nsp1-FG chains) and thin (cross-links made of single Nsp1-FG chains) segments. Video S1 shows how during simulation wild-type_ring the initially completely extended Nsp1-FG chains assume random conformations and form bundles as those seen here. Video S2 provides a three-dimensional view, reached through rotating the system in front of the viewer, of the conformation reached in simulation wild-type_ring after 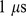, namely the conformation depicted in *(c)* and *(e)*.

For simulation wild-type_ring, Nsp1-FGs were grafted to a gold nano-ring that matches the pore dimension of the pore system designed by an experimental group [21], [29], engineered to mimic the NPC geometry. The system was simulated for 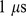 after which time the initially fully stretched Nsp1-FGs formed brush-like bundle structures (see Figure 1), the same structure as reported in an earlier study of 100-aa-long fragments of FG-domains [20]. Such structures can potentially function as a selective barrier to transport, supporting the virtual-gate model of nuclear pore gating [1], [20]. Key for the gating mechanism is that the bundles are interconnected via single Nsp1-FG chains cross-linking adjacent bundles (see Figure 1*(c)* and Figure 1*(e)*).

**Figure 2:**
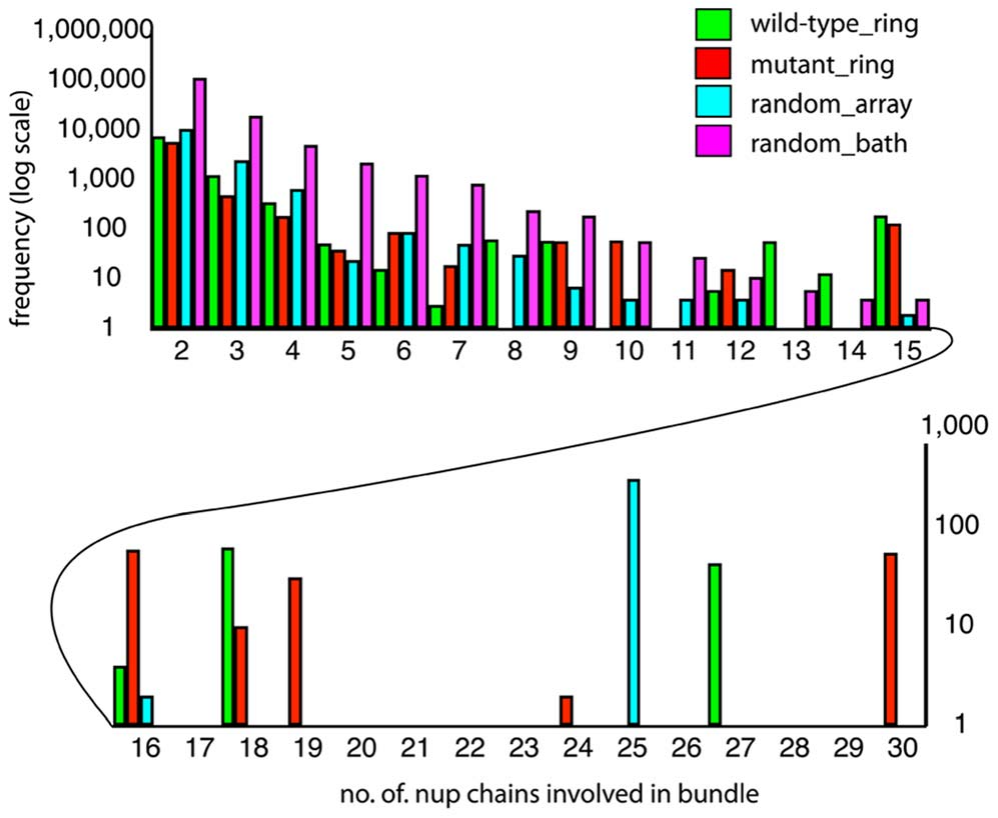
Bundle thickness distribution. Bundle thickness is determined by the number of Nsp1-FG chains involved in a bundle. Shown is here the distribution of these numbers for the simulations carried out. The frequencies with which chain numbers arise were averaged for the last 30 ns of the four 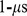 simulations wild-type_ring, mutant_ring, random_array, and random_bath. Bundles with fewer than ten Nsp1-FG chains favor a mesh-like structure, namely bundles with frequent cross-links, whereas bundles with more than ten Nsp1-FG chains exhibit brush-like structures with relatively few cross-links. Green represents the frequency distribution of Nsp1-FGs for simulation wild-type_ring, red for mutant_ring, cyan for random_array, and purple for random_bath.

We analyzed the observed brush-like bundle structures through computation of the bundle-thickness distribution. Bundle thickness is determined in terms of the number of different Nsp1-FG chains present in a bundle. For the brush-like structure as shown in Figure 1, bundles with few chains are most favored entropically, yet the bundle thickness distribution shows that there arise also thick bundles that consist of more than fourteen chains (Figure 2).

To test which role FG-repeats play in the formation of the structure shown in Figure 1, we simulated a mutant system replacing all phenylalanines and glycines in Nsp1-FG by alanines (simulation mutant_ring). The assembly structure of mutant Nsp1-FGs in the nanopore model resulting from 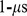 simulations is very similar to that seen for wild-type (WT) Nsp1-FGs. The similarity is also reflected in the bundle distribution shown in Figure 2 that shows a bimodal shape with thin and thick mutant Nsp1-FG bundles arising. The dynamics of Nsp1-FG assembly during the simulation and a detailed 360-degree view of the brush-like structures that resulted from the 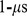 simulation are provided in Videos S1 and S2, respectively.

**Figure 3:**
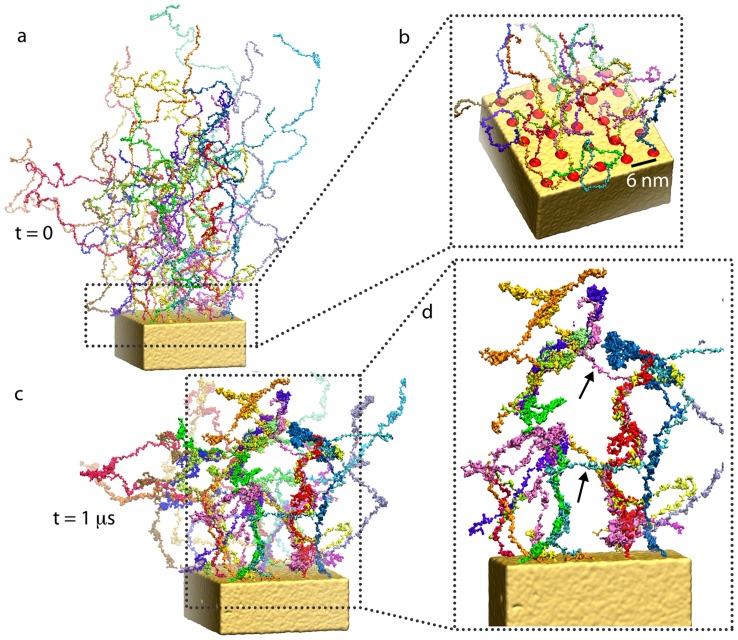
Initial and final configuration of a simulated array of wild-type Nsp1-FGs grafted to a gold substrate (simulation random_array). *(a)* Initial configuration. Shown is the 5×5 array of wild-type Nsp1-FGs grafted with their C-terminal ends to a gold substrate. The proteins are placed initially in random polymer-like conformations obtained computationally through a description of non-overlapping worm-like chains. Colors distinguish the 25 grafted wild-type Nsp1-FGs. *(b)* Close-up view of grafted ends of the Nsp1-FG chains in an array. Shown as red spheres are C-terminal ends of the Nsp1-FG chains fixed to the gold substrate, as well as the terminal segments of the Nsp1-FG chains. *(c)* Snapshot of the (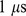) end of simulation random_array. One can see that the end-tethered, randomly placed (matching a worm-like chain model) Nsp1-FGs, shown in surface representation, form brush-like structures as in case of simulation wild-type_ring, but with a higher density of cross-links compared to the gold ring case shown in Figure 1. *(d)* Close-up view of a segment of *(c)*. The view reveals cross-linked Nsp1-FG bundles. Arrows point to cross-links between bundles formed when Nsp1-FG chains cross from one bundle to another bundle. Video S3 shows how during simulation random_array the initially completely random conformation of Nsp1-FG chains assume a mesh-like structure by cross-linking between thin bundles as those seen here. Video S4 provides a 360-degree view of the conformation reached in simulation random_array after 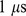, namely the conformation depicted in *(c)* and *(d)*.

The initially fully- extended and straight-chain Nsp1-FGs, simulated in [20] and in the present simulations (wild-type_ring and mutant_ring), are highly ordered at the outset. These simulations may lead to trapped and, thus, unrealistic states that are in quasi-equilibrium, calling into question if the assembly structure shown in Figure 1 is representative for the NPC interior. Therefore, we modeled in simulation random_array end-tethered Nsp1-FGs with a grid spacing of 6 nm, similar to that in the gold ring geometry, but assumed, for the initial state, random rather than straight (Nsp1-FG) conformations. The simulation involved again the full-length (609-aa) Nsp1 FG-domain, Nsp1-FG. From the present simulation resulted, nevertheless, a brush-like structure of bundles with the bundle distribution shown in Figure 2. However, the bundles formed exhibit more cross-links than seen in the earlier simulation [20] (see Figure 3). A view of the formation, during simulation random_array, of brush-like structures with cross-linked bristles and a 360-degree view of the final conformation are shown in Videos S3 and S4, respectively.

**Figure 4:**
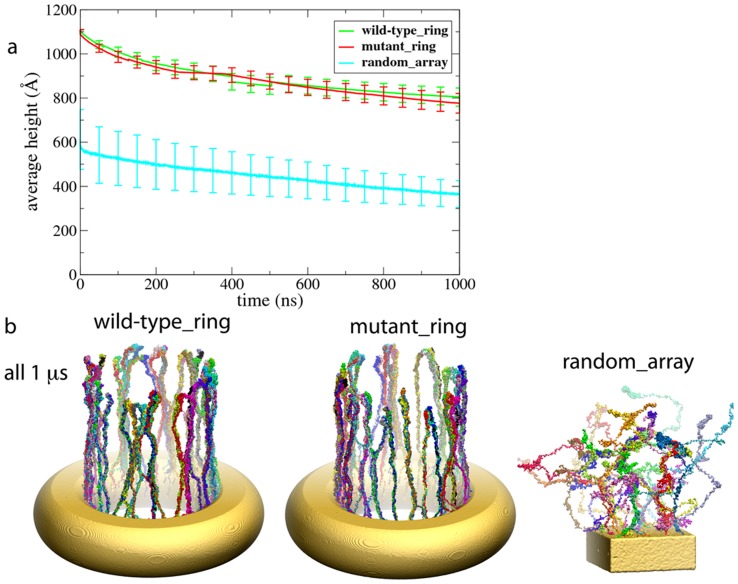
Height of Nsp1-FG chains. *(a)* Time evolution of the average height. The height is shown for 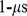 simulations for the end-tethered Nsp1-FGs in simulations wild-type_ring (green line), mutant_ring (red line), and random_array (cyan line). The heights are calculated as the average end-to-end distance in the 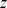-direction, the average being taken over all chains in a given simulation system. *(b)* Snapshot of the (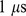) end of simulation wild-type_ring (see also Figure 1*(c)*), mutant_ring, and random_array (see also Figure 3*(c)*). Videos show how during simulation wild-type_ring, the fully extended Nsp1-FG chains form brush-like, strongly cross-linked bundles (Video S1) and how in simulation random_array, the worm-like chain Nsp1-FGs form non-brush-like bundles (Video S3). The mutated Nsp1-FG chains (FG-to- AA) arising in simulation mutant_ring form brush-like bundles with similar average brush height as seen to arise for wild-type Nsp1-FGs in simulation wild-type_ring.

The brush-like structure resulting for ensembles of closely spaced Nsp1-FGs can similarly be characterized by brush height. For the end-tethered Nsp1-FGs in simulations wild-type_ring, mutant_ring and random_array we monitored the average brush height, namely the end-to-end distance of the main-chain C*_α_* beads of the Nsp1-FG chains in the 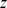-direction (Figure 4). The average was taken over all Nsp1-FG chains in the given simulation system. The Nsp1-FG chains in the final structures for both 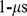 simulations, namely wild-type_ring and mutant_ring, exhibit similar average height of about 80 nm. Interestingly, we observed that in both cases the average height decayed over time in a similar fashion on the time-scales of the simulations. The Nsp1-FG chains in the final structure of the 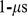 simulation random_array exhibited an average height of 60 nm.

**Figure 5:**
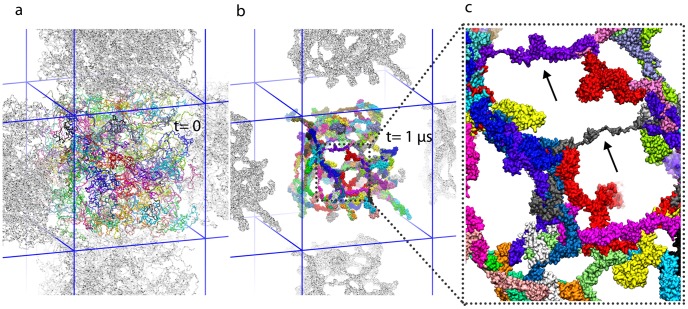
Initial and final configuration of simulated wild-type Nsp1-FGs in a solvent bath (simulation random_bath). *(a)* Initial configuration. Shown is the periodic (see simulation conditions as described in Methods) system of wild-type Nsp1-FGs, freely floating in a solvent bath (water and ions). The initial random conformations match a polymer melt modeled from worm-like chains. Colors distinguish 120 freely floating Nsp1-FG chains. Neighboring boxes in 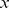
- and 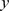
- directions are shown with the Nsp1-FGs colored in grey. *(b)* Snapshot of the (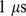) end of simulation random_bath. The Nsp1-FG chains, shown in surface representations, are seen to form a porous mesh of cross-linked Nsp1-FG bundles. *(c)* Close-up view of the structure in *(b)*. Shown is a region as marked. The view reveals a system of short bundles that are frequently cross-linked; arrows point to the cross-links between bundles. Video S5 shows how during simulation random_bath, the initially completely random Nsp1-FGs assume the final structure seen here. Video S6 provides a 360-degree view of the conformation reached in simulation random_bath after 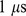, namely of the conformation depicted in *(b)*.

In order to investigate how the final structure of closely spaced Nsp1-FGs is affected by end-tethering, we carried out a simulation of initially fully disordered Nsp1-FGs freely floating in a bath (random_bath) at a protein density similar to that in random_array. These Nsp1-FGs formed again bundles (see Figure 5), but in this case with many more links between bundles (formed by single Nsp1-FGs crossing between bundles) than seen in case of simulations wild-type_ring, mutant_ring and random_array. The resulting mesh-like structure can be clearly distinguished from the brush-like structures of wild-type_ring, mutant_ring and random_array through the bundle thickness distribution shown in Figure 2 as the bundles arising in simulation random_bath exhibit at most fifteen chains per bundle. Videos S5 and S6 show the dynamics of Nsp1-FGs as seen in simulation random_bath as well as a 360-degree view of the resulting structure, respectively.

Given the different structures formed by Nsp1-FG assemblies, as they result from the present simulations and represent likely the interior of the NPC, one wonders if any specific interactions, in particular, hydrophobic FG-FG interactions, favor the structures seen. We determined, therefore, for the different types of amino acids involved in the formation of bundles how often particular amino acids arise in bundles. For simulation wild-type_ring all types of amino acids as well as FG motifs exhibit a similar propensity to be involved in bundle formation, suggesting that FGs are not particularly critical for the formation of these structures (Figure S1). This is also supported by simulation mutant_ring, in which the FG motif was replaced by alanines, and by an earlier study [20]. Moreover, all amino acids, including both hydrophilic and hydrophobic residues, are equally favorable for the brush-like structures of the wild-type_ring. Actually, no structures arising from simulations wild-type_ring, mutant_ring, random_array or random_bath exhibit particular amino acid preferences. Apparently, formation of the observed bundle structures is not sequence-specific and likely comes about through contributions from both hydrophobic and hydrophilic interactions. However, Dolker et al [30] showed hydrophilic residues in the linker regions to play a key role in the nup aggregation process and structure, whereas the role of aromatic phenylalanine residues is less dominant.

**Figure 6:**
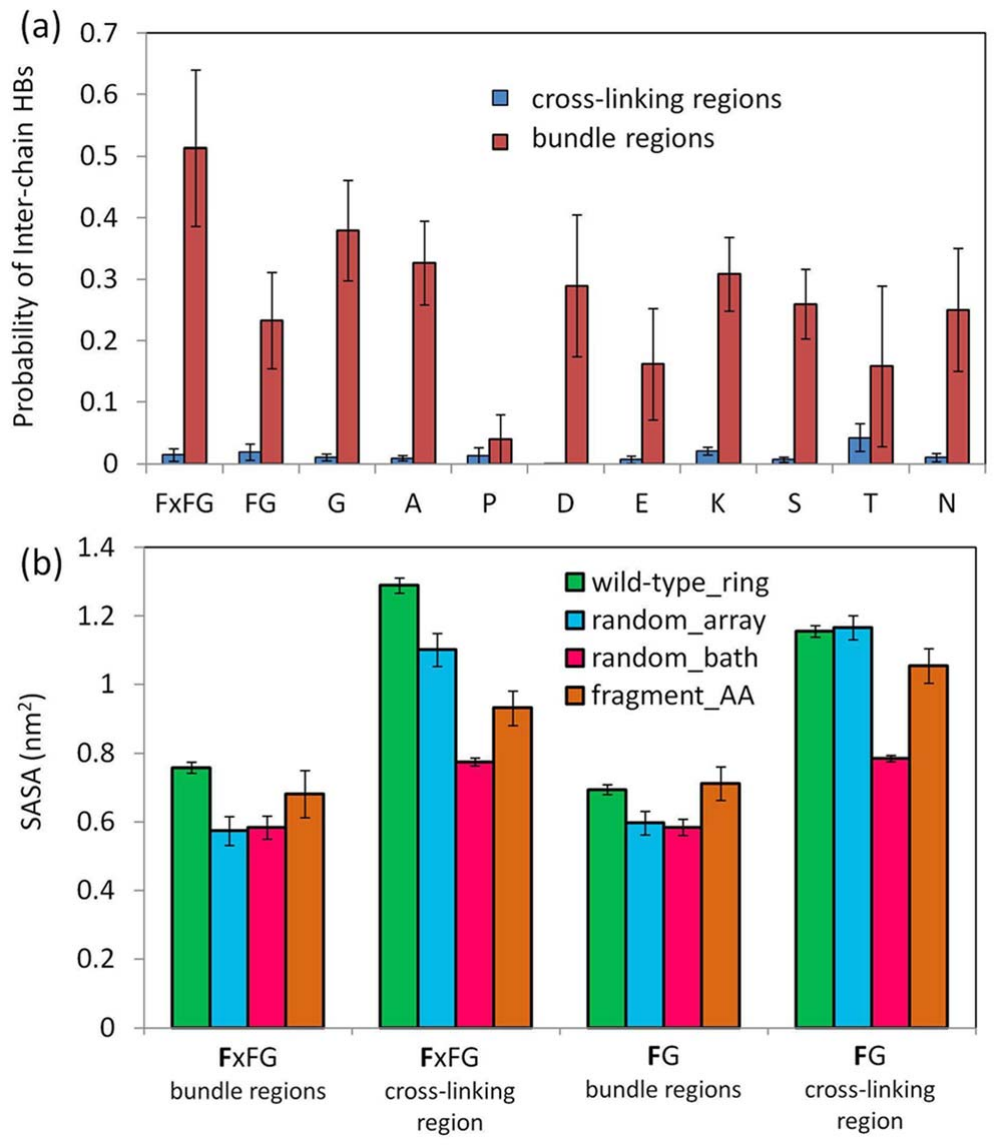
Structural features of bundle regions and cross-linking regions observed in coarse-grained and all-atom molecular simulations. *(a)* Probability of amino acids to form inter-chain backbone-backbone hydrogen bonds (HB) when located inside the bundle regions (brown bars) or inside the cross-linking regions (blue bars). Shown are the results obtained from the all-atom simulation. All amino acids located outside the bundle regions are considered to be inside the cross-linking regions. The bars labelled FG and FxFG denote the probabilities for the first phenylalanine residues in FG and FxFG motifs, respectively. *(b)* Solvent accessible surface area (SASA) of the first phenylalanine residues that are located either inside the bundle regions (labeled “bundle regions”) or inside the cross-linking regions (labeled “cross-linking regions”). SASA values from CG simulations wild-type_ring, random_array and random_bath (summarized in Table 1) are compared with SASA values from the all-atom simulation fragment_AA (Table 1) which comprises of eleven Nsp1-FG fragments of the final cross-linked bundle structure resulting from the CG simulation wild-type_ring.

To further characterize the structural details of bundles, we performed a so-called reverse CG calculation [31]–[33] on a fragment comprising cross-linked bundles arising in the final structure of simulation wild-type_ring. Such calculation assigns an all-atom (AA) model consistent with a CG model. We then employed this AA model as a starting point of a 100-ns equilibration simulation (simulation fragment_AA). These fragments of bundles increased bundle thickness during the equilibration (see Figure S2), likely due to the absence of end-tethering of proteins resulting from the fragmentation applied. Inter-chain backbone-backbone hydrogen bonds (HBs) frequently formed (∼30–50%) for most amino acids within the bundles, and played a critical role for the stability of the bundles (Figure 6a). Secondary structure analysis, on the other hand, revealed no apparent preference (<5%) for either *α*-helix or *β*-sheet formation for any amino acid. To assess the role of FG and FxFG motifs on nup assembly, we determined the solvent accessible surface area (SASA) for individual amino acids; the results are shown in Figure 6b for the FG and FxFG motifs present in a bundle region or in a cross-linking region. The SASA values from both CG and AA simulations show that both FxFGs and FGs adopt a higher degree of solvent accessibility when present in a cross-linking region than compared to SASA values arising for these motifs in a bundle region (Figure 6b); in this respect FxFG and FG motifs behave very similarly (Figure 6b).

**Figure 7:**
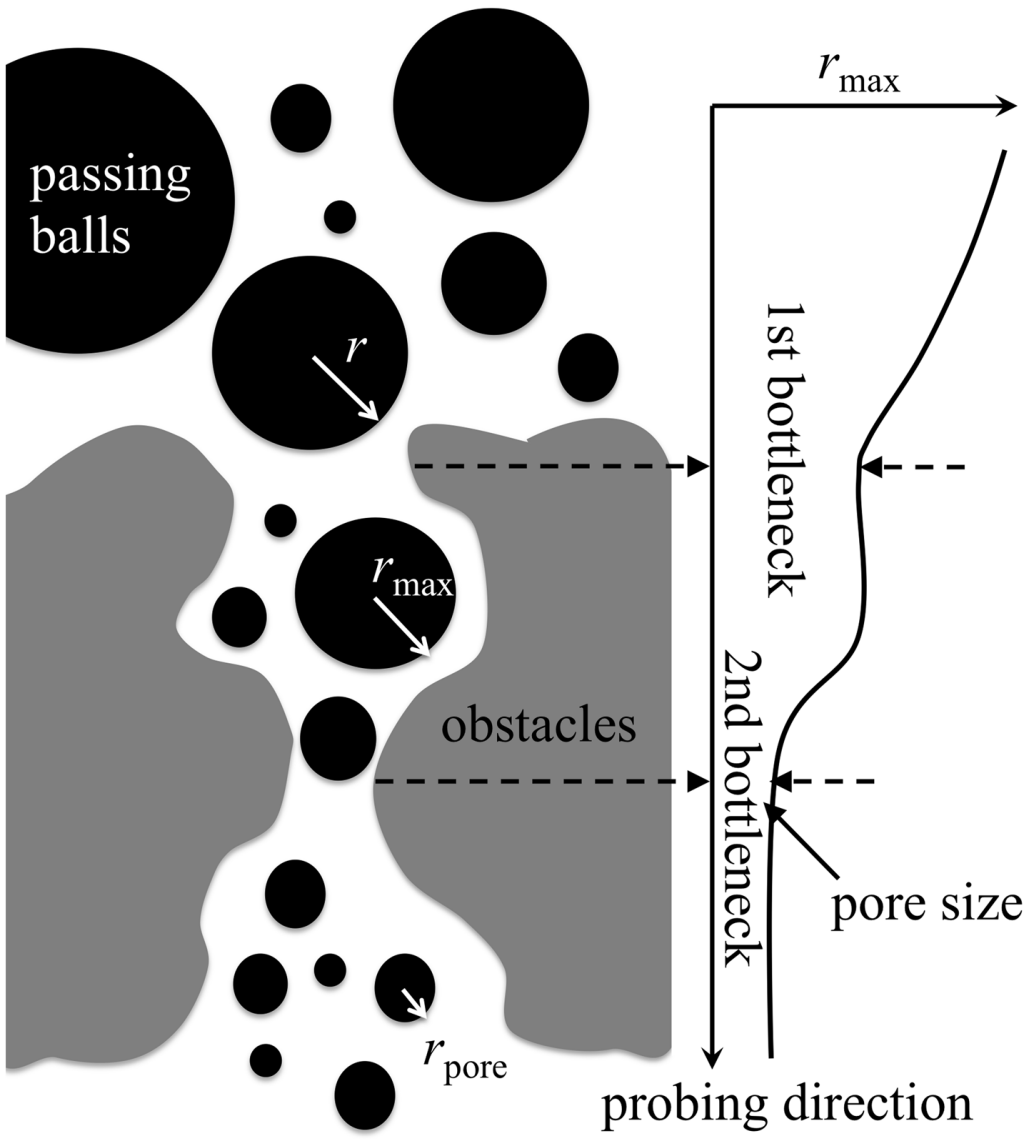
Schematic algorithm for calculating pore sizes. The figure shows a schematic depiction of the algorithm employed in calculating the pore sizes listed in Table 2. The pore size is defined through the radius of the largest spherical cargo capable of passing through the final structure resulting from a 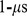 simulation of a system of Nsp1-FGs. A search for the largest cargo starts on one side of the system, the latter shown in grey. The cargo (black sphere) of a certain size moves towards the other side while the algorithm probes if the cargo can pass. The panel at right illustrates what is measured by the algorithm, namely the radius of the largest cargo that can pass through along a probing direction. As shown in this panel, there are two bottlenecks, the second smaller one of which determines how large a ball can pass through the obstacles and, therefore, characterizes the pore size of obstacles.

In order to determine in how far the structures of Nsp1-FG assemblies described above provide a barrier against the diffusion of molecules through them, we computed the size of passing molecules as described in Methods and illustrated in Figure 7. Table 2 lists for the structures resulting from simulations wild-type_ring, mutant_ring, random_array, and random_bath the average radius for the cargo molecules that can diffuse through the structures. Large pores are available for diffusive passage when Nsp1-FGs are assembled into bundle structures with few cross-links; in this case molecules with a radius as large as ∼77 Å can pass, whereas mesh-like bundle structures with many cross-links furnish only relatively small pores for molecules to pass through, namely only ones with radii smaller than 43–50 Å.

**Table 2:** Average pore size.

Name	Average pore size ± std.dev Å
wild-type_ring	77.32±0.92
mutant_ring	77.35±0.94
random_array	50.38±1.40
random_bath	43.33±1.33

The pore size is defined through the radius of the largest spherical cargo capable of passing through the final structure of a simulated system of Nsp1-FGs. The radius was determined according to the algorithm presented in Methods and illustrated in Figure 7. The average was taken over the last 30 ns of the 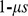 simulation for each of the systems wild-type_ring, mutant_ring, random_array, and random_bath.

Our results suggest structural properties of closely interacting Nsp1-FG proteins. We contrast these properties with the structural property of an isolated Nsp1-FG protein. The structures exhibited by a single untethered Nsp1-FG protein monitored in a 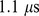 CG MD simulation showed very different features from the structures resulting from the ensembles of closely spaced Nsp1-FG proteins in simulations wild-type_ring, mutant_ring and random_array. An initially fully-extended, untethered single Nsp1-FG is found to coil up into a globule-like structure, with its radius of gyration (R*_g_*) decreasing from over 300 Å (corresponding to the initially fully-extended form) to 65 Å (corresponding to the final, coiled form) as shown in Figure S3. Similar globule-like structures were also observed in earlier studies of dynamics of individual, end-tethered short fragments of Nsp1-FGs [20].

## Discussion

The structure of the NPC central channel interior, made up of intrinsically disordered nups with FG-repeats, governs transport through the NPC. The structure formed by the assembly of tethered nups determines the size of small molecules that can passively diffuse through. The structure also interacts, likely through the FG repeats, with the transport factors that carry various cargoes through the NPC.

To characterize the structure of the channel interior, we investigated the assembly using one type of FG-nups, namely Nsp1-FG, that is present in the central channel. We studied, using CG MD simulations, the dynamics of Nsp1-FGs starting from three different initial states: tethered, straight-chain conformations; tethered, random-chain conformations; and untethered, random-chain conformations, simulating each system for 1 *µ*s. We observed in all cases the formation of brush-like bundles linked through single Nsp1-FGs crossing between bundles. This assembly structure is very similar to one seen in prior simulations of initially fully-extended, Nsp1-FG fragments (100-aa-long), that were closely grafted (2.6 nm apart compared to 6 nm-spacing in the present study) in an array-like arrangement [20]. On the other hand, a new finding of the current study is that frequency of crossings between bundles and bundle thickness depend on protein length, on the geometry of the simulated volume, on the degree of tethering, and, particularly, on the initial conformations of the proteins.

An interesting observation is that FGs may not be the only factor that plays a role in the formation of bundles. In our present mutant study (simulation mutant_ring) and that reported in [20], basically the same structures of brush-like bundles with cross-linking nups are formed as with wild-type Nsp1-FGs, suggesting that factors other than FG motifs could also contribute to the formation of bundle structures. This finding is also supported by our analysis showing that all kinds of amino acids have similar propensity to arise in the bundles formed. This finding is in contrast to a study of cross-linking arising in a hydrogel-like structure suggested for the selective phase model: in such structure low affinity inter-FG hydrophobic interactions, supposedly, are responsible for cross-linking [23], [24].

We also note that Miller et al [34] showed through EM experiments that another nup, namely Nup153, is less aggregation-prone when having its FG motifs either deleted or mutated into AG. However, owing to different nups and mutations investigated in their study and ours, we are unable to address the discrepancy between the two studies.

The FG motifs not only play a role in bundle formation but also assist in transport of TF through the NPC channel. A recent study showed that the presence of unbound and, thus, freely available FG-repeat motifs helps binding all possible sites of the TF and is required for efficient cargo transport [35]. In line with that study, a portion of the FxFG and FG motifs are found in the present study, through a SASA analysis in the framework of both CG and all-atom simulations, to be highly accessible to solvent in the cross-linking regions between the bundles. Accordingly TFs, dotted with FG binding sites, should bind to the multiple FGs arising in the cross-linking regions and, perhaps, tear the bundles apart in a zipper-like fashion.

Our interpretation, based on the structural features observed for tethered, intrinsically disordered FG-nups of the transport channel, agrees with the other views [18]–[21], [23]–[27], [36] in that the nups form characteristic quasi-stable, i.e., slowly varying, structures bearing FG motifs, with which TFs are known to interact. Thus, interaction between TF surfaces and nups is structured and cannot solely be described properly by employing the potential of mean force, as suggested by Tagliazucchi et. al, which does not include the assembly of nups in the interior of the channel [28]. Experiments employing single-molecule studies that deduced spatial density plots for TF-FG-repeat interactions have also shown that the interaction sites are not evenly distributed in the NPC; instead, they form spatial clusters inside and outside the central nuclear pore [37].

Other models have been suggested for the structures formed by nups in the NPC. For example, experiment and simulation suggest that nups aggregate as amyloid like fibers, in which *β*-sheet rich sheets form through inter-chain backbone-backbone hydrogen bonding and then through stacking of these sheets. Indeed solid-state NMR data are consistent with that such amyloid-like structures in gel-like aggregates formed by Nsp1-FGs mainly through interactions between hydrophilic residues [38]. Likewise, all-atom simulations of ensembles involving the small peptide FSFG showed that the hydrophobic residue phenylalanine can form amyloid-like aggregates [30], a feature apparently also consistent with a high-resolution EM study of nup aggregates [34]. While the MARTINI force field [39] employed in the present study is unable to model *β*-sheets, our all-atom simulations based on a reverse CG calculation as described above is capable of yielding *β*-sheet formation, and indeed resulted in considerable inter-chain backbone-backbone hydrogen bonds in the bundles, but yielded only a small degree of *β*-sheet structure. Clearly, the structural variety of actual nups aggregates in the NPC still needs to be investigated further through experimental and computational means.

**Figure 8:**
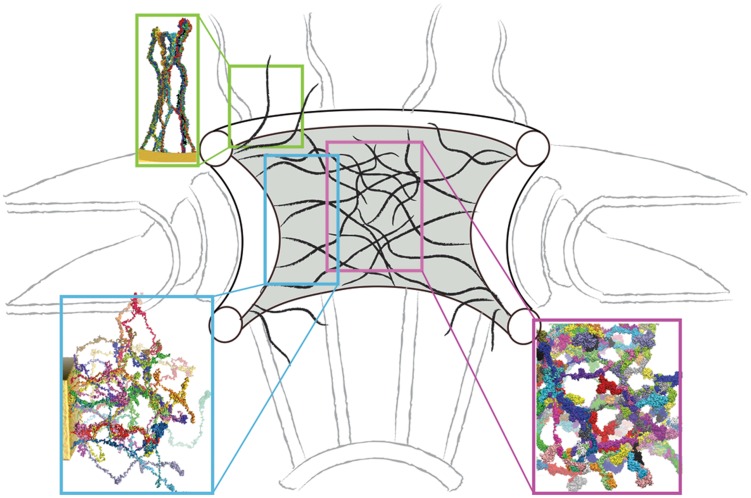
Schematic model for the structural assembly of nups in the NPC channel. The strands in black represent bundles of two or more Nsp1-FG chains. The frequency of cross-linking is higher in the central region, a feature that can be identified with the assembly of nups formed in simulation random_bath (inset in purple) characterized as a sieve-like mesh; in the periphery, brush-like bundles with less cross-linking arise and the respective structure can be identified with the ones developed in simulations random_array (inset in blue) and wild-type_ring (inset in green).

A recent model [27] for the structure inside a NPC transport channel proposed that the central region is gel-like and the periphery is brush-like. The authors postulate two zones, a central and a peripheral zone, for trafficking of molecules in and out of the NPC. Similarly, we model the structure of the entire transport channel of the NPC based on our simulation results. In our study, we did not simulate the entire region of the channel interior as this is computationally still too expensive presently. Instead, we simulated systems of Nsp1-FGs starting from different initial states and based on the results suggests a heterogenous model for the NPC central channel: (i) at the periphery, i.e., close to the scaffold, the FG nups are end-tethered to the scaffold through linker proteins; (ii) in the middle region, FG nups adopt natively disordered conformations filling up the central volume. The salient features of nups in a real NPC channel arise in our model through end-tethering of nups, protein density, random conformations for initial states, and a full-length real FG-nup found in the NPC, namely the Nsp1-FG domain. Therefore, we interpret our simulation results then to imply that the nups form different structures in different regions of the central channel (see Figure 8). The region in the periphery of the central channel, away from the center, should be represented by a system of tethered nups as in simulation random_array, where brush-like bundles with less cross-linking arise. The NPC inner diameter is about 30 nm and unstructured nups that are several hundred amino acids long span this volume of an open pore. Since tethering effects should be minimal in the central region due to the large distance from the NPC wall, where nups are actually tethered, we expect that the structure found in this region is similar to that seen in simulation random_bath, exhibiting bundles with a high degree of cross-linking forming a mesh. In this region, we observed the pore size to be 9–10 nm (Table 2) which agrees with the experimentally determined size limit for passive diffusion of small molecules.

In the present study we modeled a homogeneous system of nups consisting of only Nsp1-FGs. FG-nups in the real NPC central channel are heterogeneous; FG-nups vary across the channel volume in length, amino acid composition, FG repeat motifs (FG, FxFG and GLFG), composition and spacing of the linker regions. However, both simulation of a short, 100-aa-long fragment of Nsp1-FG in [20] and the current simulation of a full length Nsp1-FG (609-aa-long) led to the same brush-like structure model, suggesting that the interlinked brush-like structure discovered are not sensitive to varying length and type of proteins. Also, nups are known to be intrinsically disordered, interacting with each other through FG-repeats distributed throughout their entire sequences. Such disorder and interactions between FG-repeats were also captured in the present model. Therefore, we suggest that FG nup heterogeneity does not yield qualitatively different nup assembly structures.

In addition to the structural features of the NPC, it is necessary for an understanding of the transport mechanism of the NPC to examine the dynamics of assembly and conformational change of the nup proteins. Transport arises on a millisecond timescale [40], which is beyond the microsecond time scale accessible to large scale MD simulation and performed in the present study. Thus, we are unable to infer information of dynamics of the transport process from the current simulations. Instead, our simulation results should be interpreted as quasi-static pictures of dynamic assembly structures of the nups.

The major unsolved problem regarding our still limited knowledge of the NPC and NCT is how specific large cargoes with the assistance of TFs manage to pass the NPC. Clearly the NPC being one of the largest and at the same time one of the most dynamic macromolecular systems in eukaryotic cells still holds great secrets and offers opportunity for great discoveries.

Our suggestion here of the principal assembly structure of disordered nups in the NPC, even if completely true, does not imply yet how transport factors can melt the assembly structure for passage. Straightforward MD simulations, even when simplified through coarse-graining, cannot bring about answers as the systems and processes that need to be simulated are much too large and much too slow, respectively. This calamity is actually a bonus as the combination of theory, experiment, and simulation needed is intellectually more rewarding than a straightforward tour de force purely computational strategy. But in pursuing the role of transport factors one needs to be open-minded about the possibility that yet unsuspected mechanisms play a major role.

## Methods

In this section we describe the systems simulated and the molecular dynamics protocols employed in the present study. We note here that all molecular images in this article were rendered using the molecular visualization software VMD [41].

### Fully extended Nsp1-FGs on a gold nanopore

The FG-repeat domain of wild-type Nsp1, Nsp1-FG, was built from *Saccharomyces (S.) cerevisiae* Nsp1 sequence 1–609 (Swiss-Prot P14907) by using the 2004.03 release of Chemical Computing Group's Molecular Operating Environment (MOE) software. The backbone dihedral angles (phi, psi) were set to (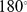, 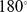) so that an unstructured straight Nsp1-FG was obtained. In case of simulation wild-type_ring, we tested this model through comparison to experiments reported in [21], [29] that have engineered, using nanotechnology, an artificial pore channel employing nuclear pore proteins inside a gold ring. The system dimension and protein density were chosen to imitate volume-wise the interior of the NPC. We reproduced the gold ring dimensions adopted in [29] and distributed over the ring tethering points holding the C-terminal ends of full-length Nsp1-FG domain proteins (609-aa-long). As shown in Figure 1, 120 fully-extended wild-type Nsp1-FG chains were grafted to the ring in three concentric rows with 6 nm spacing between adjacent Nsp1-FGs [29]. The C-terminus of the end-tethered Nsp1-FGs was modified by adding five cystine residues that remained fixed to the gold surface throughout the simulation. In [20], [21], [42], these cystine residues formed thiol linkages with the gold substrate. With the C-terminus of each chain attached to the gold-ring and the rest of the chain fully-extended in the direction shown in Figure 1, the whole system was coarse-grained and solvated with CG water in a box large enough to prevent proteins from interacting with their periodic images. A total of 100 mM NaCl was added to the water box, adjusting the relative concentrations of 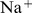 and 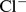 to render the whole system neutral. The resulting system simulation wild-type_ring has 15,453,214 CG beads. For the simulation mutant_ring we constructed the ring system as above and replaced all phenylalanines and glycines of the Nsp1-FGs by alanines. The resulting system described in simulation mutant_ring has 16,019,434 CG beads. Both systems were simulated for 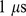 using coarse-grained molecular dynamics simulations as described below.

### Generating random conformations for Nsp1-FGs

To introduce disorder in Nsp1-FG chains, initial random Nsp1-FG conformations needed as starting points for simulations random_array and random_bath were modeled according to the widely used worm-like chain model [43]. The assignment of the random conformations proceeded in two steps. In a first step, the main-chain C*_α_* beads of Nsp1-FG constituting the protein backbone were modeled as random homo-polymers constructing the backbone from a self-avoiding walk (SAW) procedure [44]. In this procedure, the SAW is directed under two local geometric restraints, namely keeping a fixed distance of 3.7 Å between neighboring C*_α_* beads and keeping a fixed angle of 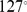 for three adjacent C*_α_* beads. The stated values are used in the MARTINI force field [39] for polypeptide chains with coil conformations. Any two C*_α_* beads were considered to be in close contact if their distance is shorter than 8 Å. We discarded any conformations of backbone chains with beads in close contact within one chain (intra) or between different chains (inter). In the second step, CG side-chains of amino acids were grafted on to the resulting backbone chains.

The geometries of the side-chains and of the gold nano-particles were modeled with standard parameters in the MARTINI force field [39], [45]. The MARTINI CG force field has been extensively optimized in order to correctly model partition between water and non-polar solvent for amino acids including phenylalanine, glycine and alanine that are of interest in the current study. Moreover, multi-site representation of large amino acids is employed in MARTINI in order to realistically model special geometric features, such as aromatic rings in phenylalanine, which are essential for side-chain interactions. In the past few years, the MARTINI force field has been successfully applied in numerous studies of peptide and peptide-lipid interactions, as well as in studies of the assembly of micelles and bilayers around membrane proteins [46]. However, due to lack of treatment of backbone-backbone hydrogen bond interactions, the MARTINI force field is unable to model formation of certain type of structures, such as 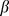-sheets.

### Random Nsp1-FGs end-tethered on a 2D array

For modeling end-tethered Nsp1-FGs in simulation random_array, we employed again for the construction of a random initial state the worm-like chain model described above and chose the last five C*_α_* beads of cysteine residues 610–614 as the starting points for the SAW. In the experimental systems [21] nups are tethered to the gold substrate by means of thiol bonds to cysteine residues added to the C-terminus. In order to be consistent with the description adopted for the ring-like geometry, the five C*_α_* beads of each Nsp1-FG chain were stretched toward the 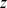-direction and placed on a 5×5 grid in the 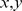-plane, with a grid spacing of 6 nm. The full-length worm-like chain was then modeled as described above, such that 25 Nsp1-FGs were placed as shown in Figure 3. The whole system was coarse-grained according to the MARTINI force field [39], [45] and solvated with CG water. The system was then ionized with 100 mM NaCl, adjusting again the relative concentrations of 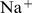 and 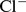 to render the whole system neutral. The resulting simulation random_array involves 1,097,433 CG beads. The system was simulated for 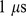 as described below.

### Freely floating random Nsp1-FGs in a bath

For simulation random_bath, the first three C*_α_* beads of each Nsp1-FG chain, treated as a rigid body, were chosen as starting points. They were randomly placed in simulation boxes with random orientations. The full-length random conformation Nsp1-FGs were then modeled as worm-like chains as described above, with 120 self-avoiding Nsp1-FGs being placed in a box of volume 725 Å×725 Å×725 Å to match the concentration of Nsp1-FGs as in the simulations wild-type_ring and random_array (see Figure 5). The whole system was coarse-grained according to the MARTINI force field [39] and solvated in a CG water box. 100 mM NaCl was then added to the sytem, adjusting the relative concentrations of 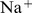 and 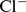 to render the whole system neutral. The resulting simulation random_bath involved 3,091,910 CG beads. The system random_bath was simulated for 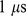 as described below.

### Reverse coarse graining

Structures resulting from CG simulations can be “reverse-coarse-grained” to obtain corresponding all-atom (AA) structures, where each amino acid can be mapped back to an AA representation by replacing the CG beads by all the atoms it represents. The method is detailed in [31]–[33]. In the present study, CG MD was employed to extend the simulation time scale to microseconds while AA MD was used to refine a segment of the final structure arising in simulation wild-type_ring to investigate the chemical details of the final brush-like structures. For the AA simulation fragment_AA, eleven Nsp1-FG chains, namely, L17(275–507), L18(275–523), L19(293–513), M21(302–544), M22(316–545), M23(317–548), M24(326–546), N26(328–579), N27(336–579), N28(332–570) and N29(345–581) of CG simulation wild-type_ring were included; the numbers in parenthesis are the residue numbers for amino acids of the respective chains included, having been selected for exhibiting a cross-link. The CG representation of the stated Nsp1-FG bundle segments, after being mapped back to an AA representation, were solvated into an AA water box. A total of 100 mM NaCl was then added to the water box, adjusting the relative concentrations of 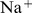 and 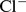 to render the system neutral. The resulting box has a volume of 123 Å×198 Å×414 Å and includes 967,595 atoms. The resulting AA structure was simulated for 100 ns as described below.

### Simulation protocol

Coarse-grained (CG) molecular dynamics simulations were performed based on the MARTINI model for proteins [39] in NAMD 2.9 [47]. For use of the MARTINI force field we adapted the GROMACS switching function for the LJ potential and a shifting function for the Coulomb potential. Non-bonded interactions were cut off at 12 Å, with shifting throughout the interaction range for electrostatic interactions and beginning at 9 Å for vdW interactions, implementing a smooth cut-off. Simulations were performed using a 10 fs timestep. Pair lists were updated at least once every ten steps, with a 14 Å pair list cut-off. In all cases we performed Langevin dynamics with a damping coefficient of 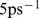. A constant pressure of 1 atm was maintained with a Nosé-Hoover Langevin piston [48], using a piston period of 2000 fs and a decay time of 1000 fs. All systems were allowed to equilibrate as follows: first, the system was energy minimized for 5000 steps and molecular dynamics was performed for 2 ns in an NVT ensemble (T = 300 K). The resulting system was then simulated for 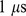 assuming an NPT ensemble.

The all-atom simulations were performed using NAMD 2.9 [47]; non-bonded interactions were cut off at 12 Å, with a switching function beginning at 10 Å and implementing a smooth cutoff. The simulations involved multiple timestepping, with a base timestep of 1 fs, short-range interactions calculated every step, and long-range electrostatics every two steps. Electrostatic forces were evaluated through the particle-mesh Ewald method [47] with a grid density of 1.0 Å^−3^. The AA system was first energy minimized for 5000 steps and then was simulated for 100 ns assuming an NPT ensemble (T = 300 K). Periodic boundary conditions were assumed for all (CG and all-atom) simulations.

### Analysis of bundles

As described in Results, the simulated Nsp1-FGs formed strands that we refer to as bundles. We define a bundle as a linearly arranged cluster of multiple parallel Nsp1-FG chains. In such a cluster, every amino acid in a segment of one Nsp1-FG chain is in contact with at least one amino acid of another Nsp1-FG chain. In order to provide a quantitative characterization of these bundles we applied graph theory to identify bundle segments in a given configuration of the Nsp1-FG chains. In this approach, each amino acid is represented by a node in a graph. If the distance of two amino acids A and B from different chains is shorter than 6 Å, the corresponding nodes in the graph are connected with an edge. In addition, the nodes for amino acids adjacent in sequence to the two amino acids A and B forming an edge are considered to be connected to the nodes for amino acids A and B. Therefore, bundle segments can be identified by examining connected components in a graph as constructed above. For this purpose we employed the breadth-first search algorithm [49].

Thickness of a bundle is determined as the number of different Nsp1-FG chains that belong to the bundle. Another bundle characteristic determined here is the fraction of each type of amino acid involved in a bundle.

### Pore characterization

Nsp1-FG polymers self-assemble, as shown in Results, into network structures. In order to assess how these structures are related to gating in the NPC we determine the size limit of spherical particles being capable geometrically to pass through the structures. This size limit defines the pore size, namely as the largest possible radius of passing particles. Figure 7 illustrates how the pore size was determined by us algorithmically. Starting from one side of the network structure, spheres of various sizes are moved towards the other side. The analysis was applied to the final structures resulting from simulations random_array, random_bath, wild-type_ring, and mutant_ring; in the case of the latter two simulations structures are formed with Nsp1-FGs arranged in a ring-like arrangement. In these cases, the spheres were moved radially from the inside of the Nsp1-FG ring structure to its outside.

We implemented the above analysis through an algorithm in which the simulation box with the network structure was mapped into a cubic lattice. We then determined for each grid point, 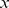, in the lattice the radius 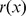 of the largest sphere that could be placed on this grid point without sterical clash with any Nsp1-FG chain; this radius property was calculated as the shortest distance between the grid point and protein beads. A sphere is considered capable of moving in the lattice along a given pathway, if the size of the sphere does not exceed the bottleneck of the pathway, i.e., the smallest 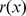 of the grid points on the pathway. There could be multiple pathways for a sphere to permeate from one side to the other side, each pathway having its own bottleneck. To characterize the largest sphere that could permeate the network structures we search for the pathway for which the bottleneck is the widest among all the possible pathways. The pathway search was performed using Dijkstra's algorithm [50]. To illustrate a typical outcome of the pore analysis, we show in Video S7 a 360-degree view of all possible pores identified in the final structure resulting from simulation random_array.

## Supporting Information

Figure S1
**Propensity for certain amino acids to be involved in the formation of bundles.** The probability for different kinds of amino acids to be involved in the formation of bundles as determined from an average over the last 30 ns of the 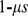 simulations wild-type_ring (green), mutant_ring (red), random_array (cyan), and random_bath (purple). G^*^ refers to glycines and F^*^ refers to the phenylalanines that are not included in FGs of FG-repeat motifs.(TIF)

Figure S2 **Initial and final configuration of an all-atom simulation of part of the final configuration of simulation wild-type_ring (simulation fragment_AA).** *(a)* Initial configuration of the all-atom model, which is a part of the large CG model as shown in Figure 1*(e)* (see Methods for how the system is reverse coarse-grained). *(b)* Final configuration after 100- ns all-atom simulation. The fragments coming from different protein chains in the original CG model are shown in different colors.
(TIF)

Figure S3
**Coiling of an initially fully-extended, untethered Nsp1 segment.** The time evolution of the radius of gyration (R*_g_*) is shown for a 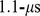 CG simulation.(TIF)

Video S1 **This video presents the dynamics of simulated wild-type Nsp1-FGs grafted to a gold ring (simulation wild-type_ring).** The colors distinguish 120 wild-type Nsp1-FGs grafted on the ring in three concentric rows. The video shows how during the simulation the end-tethered, fully-extended, wild-type Nsp1-FGs assume quickly random conformations and form brush-like bundles (video corresponds to Figure 1).
(MP4)

Video S2
**This video provides a 360-degree view of the final conformation reached after** 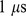 **in simulation wild-type_ring.** The video reveals the brush-like bundles formed by Nsp1-FG chains. One can also see that few of these bundles cross-link (video corresponds to Figure 1c and Figure 1e).(MP4)

Video S3 **This video presents the dynamics of an array of wild-type Nsp1-FGs grafted to a gold surface (simulation random_array).** The colors distinguish the 25 wild-type Nsp1-FGs simulated. The video shows how during the simulation the chains form brush-like structures. The bundles formed exhibit more cross-linking between themselves than seen in case of simulation wild-type_ring (video corresponds to Figure 3).
(MP4)

Video S4
**This video provides a 360-degree view of the final conformation reached after** 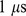 **in simulation random_array.** The video reveals the brush-like bundles formed by Nsp1-FG chains. One can also see that the bundles cross-link rather frequently (video corresponds to Figure 3c and Figure 3d).(MP4)

Video S5 **This video shows the dynamics of simulated, initially random, wild-type Nsp1-FGs, freely floating in a solvent bath (water and ions) (simulation random_bath).** The colors distinguish the 120 wild-type Nsp1-FGs simulated. The video shows how during the simulations the untethered, initially random chains form brush-like structures with a degree of cross-linking much higher than in case of simulations wild-type_ring and random_array (video corresponds to Figure 5).
(MP4)

Video S6
**This video provides a 360-degree view of final conformation reached after** 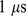 **in simulation random_bath.** The video reveals the bundles formed by Nsp1-FG chains. The bundles are cross-linked with high frequency, in fact, more frequently than either in case of simulation `wild-type_ring or simulation random_array (video corresponds to Figure 5c and Figure 5d).(MP4)

Video S7 **This video provides a 360-degree view of all pores in the simulated array of Nsp1-FG tethered to a plane.** Shown in blue are Nsp1-FG proteins that form brush-like structures interconnected with cross-links. Shown in red are the pores that can accommodate spherical objects with diameters greater than 7 nm. The pores were identified by means of the algorithm described in Methods. The narrow regions connecting wide regions correspond to the bottlenecks for passive diffusion of molecules.
(MP4)
