# Supplementary figures and images for: Assembly of Nsp1 Nucleoporins Provides Insight into Nuclear Pore Complex Gating
Source: PLoS Comput Biol. 2014 Mar 13;10(3):e1003488. doi: 10.1371/journal.pcbi.1003488 (PMC3952814; doi:10.1371/journal.pcbi.1003488)

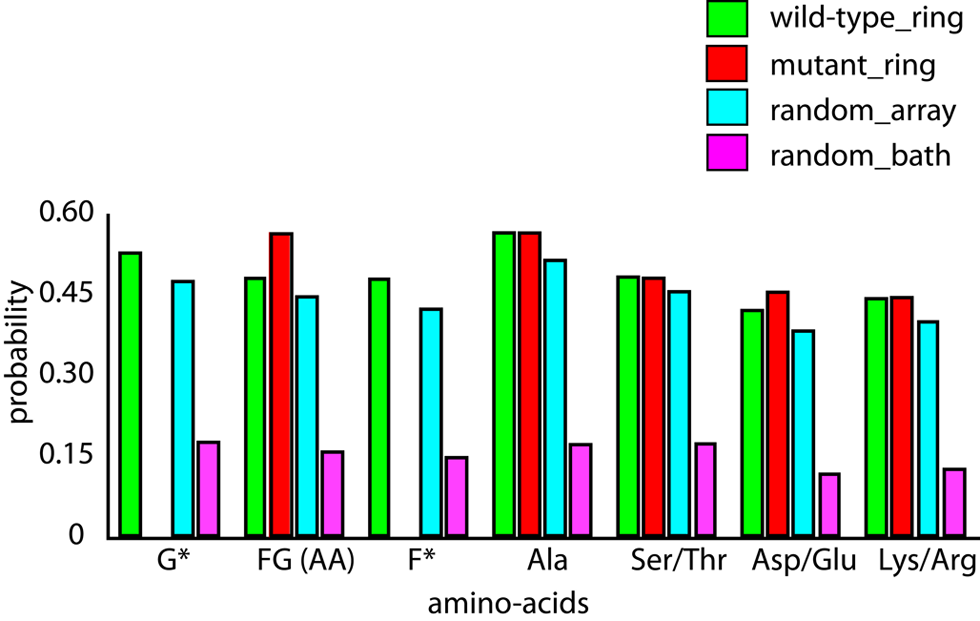

Supplement: Figure S1 — Propensity for certain amino acids to be involved in the formation of bundles. The probability for different kinds of amino acids to be involved in the formation of bundles as determined from an average over the last 30 ns of the simulations wild-type_ring (green), mutant_ring (red), random_array (cyan), and random_bath (purple). G* refers to glycines and F* refers to the phenylalanines that are not included in FGs of FG-repeat motifs. (TIF) [file pcbi.1003488.s001.tif]

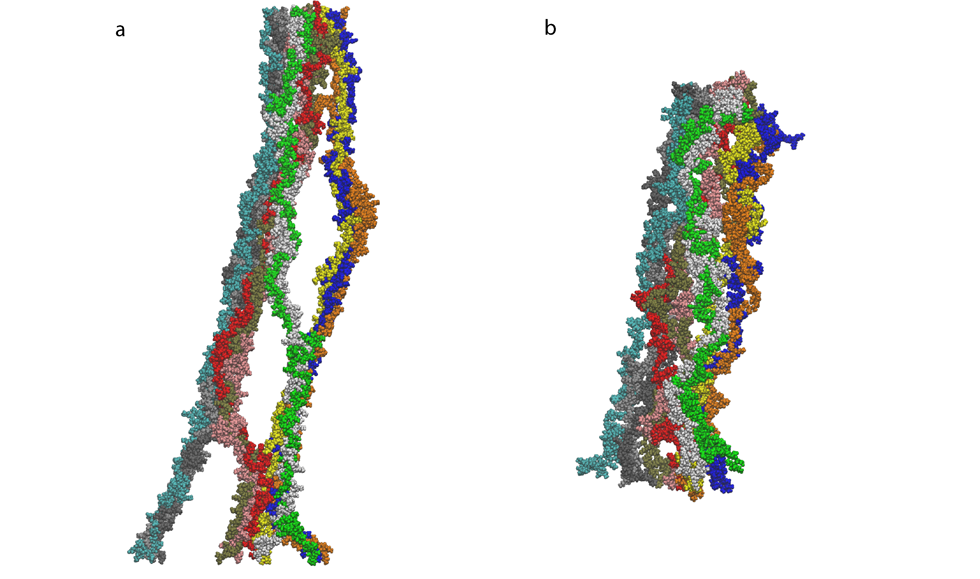

Supplement: Figure S2 — Initial and final configuration of an all-atom simulation of part of the final configuration of simulation wild-type_ring (simulation fragment_AA). (a) Initial configuration of the all-atom model, which is a part of the large CG model as shown in Figure 1(e) (see Methods for how the system is reverse coarse-grained). (b) Final configuration after 100- ns all-atom simulation. The fragments coming from different protein chains in the original CG model are shown in different colors. (TIF) [file pcbi.1003488.s002.tif]

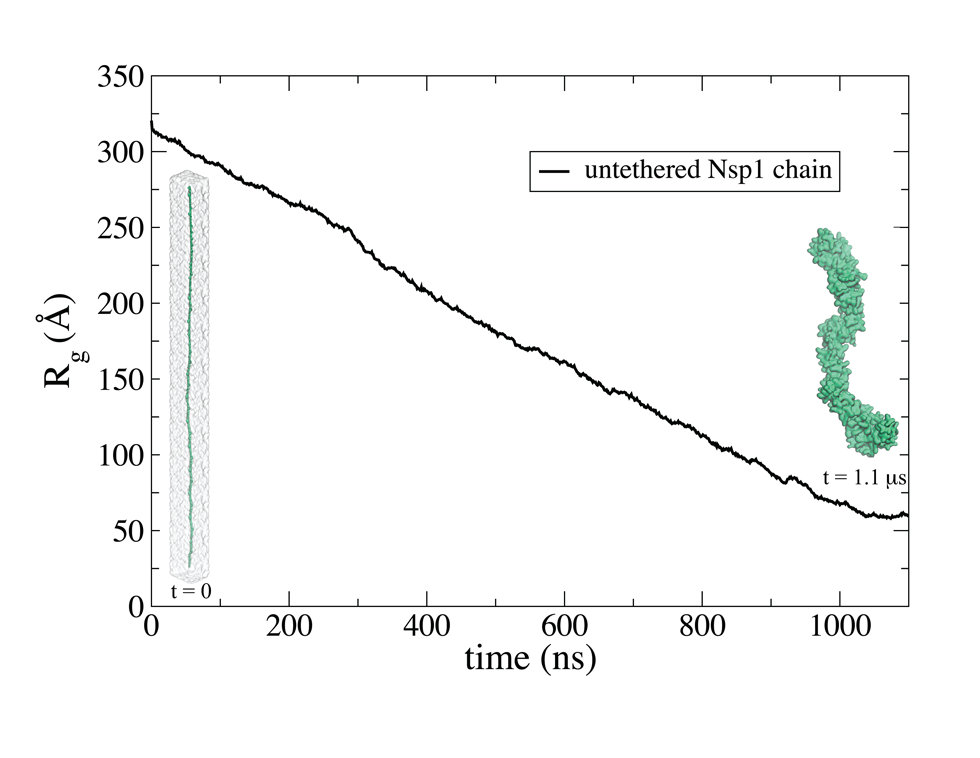

Supplement: Figure S3 — Coiling of an initially fully-extended, untethered Nsp1 segment. The time evolution of the radius of gyration (Rg) is shown for a CG simulation. (TIF) [file pcbi.1003488.s003.tif]
